# Effects of N-butanol extract of *Amygdalus mongolica* on rats with bleomycin‐induced pulmonary fibrosis based on metabolomics

**DOI:** 10.1590/1414-431X2023e13045

**Published:** 2023-11-03

**Authors:** Chen Gao, Yingchun Bai, Hongbing Zhou, Hongyu Meng, Tong Wu, Wanfu Bai, Jia Wang, Liya Fan, Yuxi Yang, Hong Chang, Songli Shi

**Affiliations:** 1 Department of Pharmacy, Baotou Medical College, Baotou, China; 2 The Second Affiliated Hospital of Baotou Medical College, Baotou, China; 3 Dongzhimen Hospital, Beijing University of Chinese Medicine, Beijing, China; 4 Institute of Bioactive Substance and Function of Mongolian Medicine and Chinese Materia Medica, Baotou Medical College, Baotou, China

**Keywords:** Amygdalus mongolica, N-butanol extract, Pulmonary fibrosis, Metabolomics, TGF-β_1_

## Abstract

Pulmonary fibrosis (PF) is a major public health issue with limited treatment options. As the active ingredient of the n-butanol extract of *Amygdalus mongolica* (BUT), amygdalin inhibits PF. However, its mechanisms of action are unclear and need further verification. Therefore, the purpose of the present studies was to investigate the anti-fibrotic effects of BUT on PF by serum metabolomics and the transforming growth factor β (TGF-β) pathway. Sixty male Sprague-Dawley rats were randomly divided into control, untreated PF, prednisone-treated (5 mg/kg), and BUT-treated (1.75, 1.25, 0.75 g/kg) groups, and the respective drugs were administered intragastrically for 21 days. The serum metabolomics profiles were determined by ultra-performance liquid chromatography quadrupole time-of-flight mass spectrometry (UPLC-QTOF/MS) and metabolism network analysis. The expression of TGF-β1, Smad-3, Smad-7, and α-smooth muscle actin (α-SMA) was measured using a real-time polymerase chain reaction in the lung tissue. BUT significantly alleviated fibrosis by reducing the mRNA expressions of TGF-β1 (from 1.73 to 1.13), Smad-3 (from 2.01 to 1.19), and α-SMA (from 2.14 to 1.19) and increasing that of Smad7 (from 0.17 to 0.62). Twenty-eight potential biomarkers associated with PF were identified. In addition, four key biomarkers were restored to baseline levels following BUT treatment, with the lowest dose showing optimal effect. Furthermore, *A. mongolica* BUT was found to improve PF by the pentose phosphate pathway and by taurine, hypotaurine, and arachidonic acid metabolism. These findings revealed the mechanism of *A. mongolica* BUT antifibrotic effects and metabolic activity in PF rats and provided the experimental basis for its clinical application.

## Introduction

Pulmonary fibrosis (PF) is a chronic malady characterized by inflammatory cell infiltration, fibroblast proliferation, and excessive extracellular matrix deposition (1). The pathological change during the initial stage of PF is inflammation, which is reversible. Therefore, early prevention and diagnosis of the disease are crucial, but currently, there are no effective therapeutic strategies to reverse PF. Nintedanib and prifenidone can slow the progression of PF, but they are expensive and have poor outcomes. Hence, it is urgent to investigate the molecular mechanisms and find novel and effective treatments for PF (2).

Traditional Chinese medicine (TCM) is receiving increasing attention owing to its structurally complex compounds and multiple pharmacological activities (3). TCMs that improve lung energy (Qi) and reduce phlegm have been reported to improve oxidant-antioxidant imbalance, delay pulmonary fibrosis processes, and improve cough, dyspnea, and other symptoms (4). *Amygdalus mongolica* (Maxim.) Ricker is drought-tolerant and unique to the Alexa desert species in the Mongolian plateau. The seeds of *A. mongolica* are used as ‘Yu Li Ren' (5) (also known as *Pruni semen*) in TCM. As a TCM plant, Yu (5) is commonly used to relieve cough and dissolve phlegm, treating dry cough and bronchitis. *A. mongolica* has been reported to contain various medicinal ingredients, such as unsaturated fatty acids, amygdalin, flavonoids, and alkaloids (6). Amygdalin is one of the active constituents of *A. mongolica* n-butanol extract (BUT) (7,8) and a raw cyanogenic glycoside compound with apoptosis-inducing properties, which is widely used in the treatment of different cancer types and organ fibrosis, such as lung, kidney, and liver fibrosis (7,9,10). Our previous studies have demonstrated that, in addition to its lipid-regulating and anti-lipid peroxidation effects, BUT has been speculated to possess a polarity site that effectively improves lung, liver, and kidney fibrosis (11-13). However, the role of BUT in PF pathogenesis remains unclear.

It has been reported that transforming growth factor-beta (TGF-β)-associated signaling pathways play an important role in lung fibrosis, which could be associated with PF pathogenesis (14). TGF-β is a major pro-fibrotic cytokine, and the TGF-β signaling pathway is involved in the development and progression of PF. Selective blockade of the TGF-β_1_/Smads pathway is a key strategy for anti-fibrotic therapy (15). The recent development in the application of TCM to interfere with the TGF-β_1_/Smads signaling pathway and thereby improve fibrosis has become a research hotspot owing to its unique advantages.

In recent years, following the development and application of high-throughput technologies, scholars have devoted themselves to the study of various biological markers associated with PF in the hope that susceptibility and effective biomarkers can be used to screen high-risk groups in addition to their use in identifying early biological effects, tissue function changes, disease development, diagnosis, or reversal of PF in the early stages of the disease (16). Metabolomic analysis is used to systematically diagnose diseases by identifying metabolic networks and biomarker groups. Its ability to comprehensively reflect the changes in the metabolites of an organism from a healthy to a diseased state, thus revealing the process of disease development, coincides with the holistic view of TCM.

This study aimed to investigate the effects of BUT on bleomycin-induced PF metabolites and the TGF-β_1_/Smads signaling pathway in rats using qRT-PCR and metabolomic analyses.

## Material and Methods

### Drugs and animals

*A. mongolica*, harvested from Yablai Gobi, Alashan, in 2018, was identified by Professor Shi Songli of Baotou Medical College as the dried mature seeds of Mongolia almond. A total of 60 specific pathogen-free (SPF) male Sprague-Dawley rats (180±20 g) were obtained from the Department of Medicine Laboratory Animal Science, Peking University (license No. SCXK (Beijing) 2017-0005). Bleomycin hydrochloride was administered via injection (Nippon Chemi-Con Co., No. 540402, Japan), whereas prednisone acetate was administered in the form of tablets (Zhejiang Xianju Pharmaceutical Co., Ltd., No. 140905, China) to the rats.

### Reagents and instruments

Reagents and instruments used were hematoxylin and eosin (H&E), Masson staining kit (Nanjing Jiancheng Institute of Biological Engineering, China); mRNA extraction reagent (TRIzol, Thermo Fisher Scientific, USA); reverse transcription kit (RevertAid First Strand cDNA Synthesis Kit, Thermo Fisher Scientific, USA); quantitative PCR reagent (RealSYBR Mixture, Beijing Kangwei Century Biotechnology Co., Ltd., China); PCR primers (Ningxia KonoJiahua Bioengineering Co., Ltd., China); CX31 microscope (Olympus, Japan); fully automated biochemical analyzer (AU640, Olympus); enzyme marker (Thermo Fisher Scientific Co. Ltd., USA); Model 7500 RT-PCR instrument (AB, USA); NanoDrop 2000 microspectrophotometer (Thermo Fisher Scientific); ultra-high-performance liquid chromatography (ExionLC, Sciex, USA); high-resolution mass spectrometry (Triple TOF 5600, Sciex); centrifuge (Heraeus Fresco17, Thermo Fisher Scientific); scale (BSA124S-CW, Sartorius, Germany); water purifier (Min-Chu D24 UV, Merck Millipore, Germany); column (ACQUITY UPLC HSS T3 1.8 μm 2.1×100 mm, Waters, USA).

### Preparation of BUT

The seeds of *A. mongolica* were peeled and crushed. Solutions of 95 and 70% ethanol (analytical reagent (AR) grade) were used as extraction solvents. The conditions of the extraction were as follows: temperature, 70°C; solid-liquid ratio, 1:10; time, 2 h. The combined extracts were concentrated under pressure to obtain a brown ethanol extract that was partitioned with water and three organic solvents with different polarities [petroleum ether (PE) < ethyl acetate (EA) < n-butanol (BU) (all AR purity)] to obtain the PE and EA extracts and BUT, respectively (17). Furthermore, the extract was concentrated under reduced pressure to obtain the corresponding yield of BUT (g) (27.95% yield), which was then dissolved in 0.5% sodium carboxymethylcellulose to obtain the required configuration for the gavage of the experimental rats (paste yield = mass of infusion / mass of seed kernels x 100%) (17,18).

### Animal model and groups

Rats were randomly divided into the control (CON; n=10), model (MOD; n=10), prednisone (PED; n=10), high-dose BUT (BUT-H; n=10), medium-dose BUT (BUT-M; n=10), and low-dose BUT (BUT-L; n=10) groups. The rats in the MOD, PED, and BUT groups underwent a single tracheal instillation of bleomycin to establish a model of PF (19). The CON group was instilled with saline, whereas the CON and MOD groups were given daily gastric irrigations of 5 mL/kg of 0.5% sodium carboxymethylcellulose. The dosage of PED was 5 mg/kg and that of BUT-H, BUT-M, and BUT-L was 1.75, 1.25, and 0.75 g/kg per day, respectively, given by gastric irrigation for 4 weeks. The experimental protocol was approved by the Medical Ethics Committee of Baotou Medical College (Approval number: 20170315).

### Specimen collection

Twenty-four hours after the last dose, the animals were weighed and anesthetized via intraperitoneal injection of 3% pentobarbital. Blood was drawn from the abdominal aorta and centrifuged at 1748.25 *g* for 10 min at 4°C. The serum was collected and stored at −80°C for biochemical and metabolomic analyses. Part of the left kidney lobe was cut into 1-cm^3^ pieces and fixed in 10% paraformaldehyde solution for H&E and Masson staining, and the rest was frozen at −80°C for qRT-PCR (6).

### Histological examination

The tissues were processed and embedded in paraffin and the sections obtained were stained with H&E and Masson staining for subsequent histological evaluations. Lung damage index or fibrosis was evaluated based on the Ashcroft histopathology scoring criteria (20).

### qRT-PCR

Total RNA was extracted from lung tissues using the TRIzol method, and total RNA concentration in each sample was measured using a NanoDrop 2000 microvolume spectrophotometer. qRT-PCR was performed following the instructions of a qRT-PCR kit. The primer sequences (Table 1) were synthesized, and the relative expression was normalized using rat actin. Gene transcript levels were calculated using the 2^-ΔΔCT^ method.

**Table 1:** Primers used for real time-polymerase chain reaction.

Gene	Forward (5′-3′)	Reverse (5′-3′)
Rat actin	CCCATCTATGAGGGTTACGC	TTTAATGTCACGCACGATTTC
TGF-β1	CGCAACAACGCAATCTATG	ACCAAGGTAACGCCAGGA
Smad3	CCAGTGCTACCTCCAGTGTT	CTGGTGGTCGCTAGTTTCTC
Smad7	GGCTTTCAGATTCCCAACTTC	CGCCATCCACTTCCCTTGT

### Ultra-performance liquid chromatography quadrupole time-of-flight mass spectrometry (UPLC-QTOF/MS)

In each group, six serum samples were randomly selected for UPLC-QTOF/MS analysis. The quality control (QC) sample was prepared by mixing sample extracts and analyzing one in every 10 samples. The mobile phase was composed of acetonitrile (A) and water (B), each containing 0.1% formic acid. The gradient for the serum sample was 2-100% A for 15 min. The injection volume for each sample was 4 µL. The column temperature and flow rate were maintained at 35°C and 0.4 mL/min, respectively.

The MS conditions were as follows: ion source Gas1, 60 psi; ion source Gas2, 60 psi; interface heating temperature, 650°C; ion spray voltage, 500 V (ESI^+^), 4500 V (ESI^-^). MS data were collected in the IDA mode. The TOF quality scan range was 60-1200 Da and was completed within 150 milliseconds, with a total cycle time of 0.56 s. A 40-GHz four-anode/channel multichannel TDC detector was used to monitor the scans, and the pulse frequency was set at 11 kHz and averaged over four scans for correction (6).

### Data analyses

The raw data processing and candidate identification were performed following previously reported studies (21). Principal component analysis (PCA) and partial least squares discriminant analysis (PLS-DA) were used to visually analyze cluster results. Potential biomarkers were screened using the values of variable importance of projection (VIP) and *t*-tests. The metabolic pathways of differentially altered metabolites were analyzed using MetPA (MetaboAnalyst software 4.0, Canada). Metabolites in these pathways were selected as key biomarkers for PF, with biomarker levels analyzed using receiver operating characteristics (ROC) via GraphPad Prism 5 (USA) to assess diagnostic power. Network analysis of the identified biomarkers was conducted based on the Kyoto Encyclopaedia of Genes and Genomes database (6).

SPSS17.0 (IBM, USA) was used to analyze the experimental data. One-way analysis of variance (ANOVA) followed by Tukey's *post hoc* test were used for group comparisons, and the differences between the groups were analyzed using Student's unpaired *t*-test. The experimental data are reported as means±SD, and P-values <0.05 were considered statistically significant.

## Results

### Histopathological analysis

**Figure 1:**
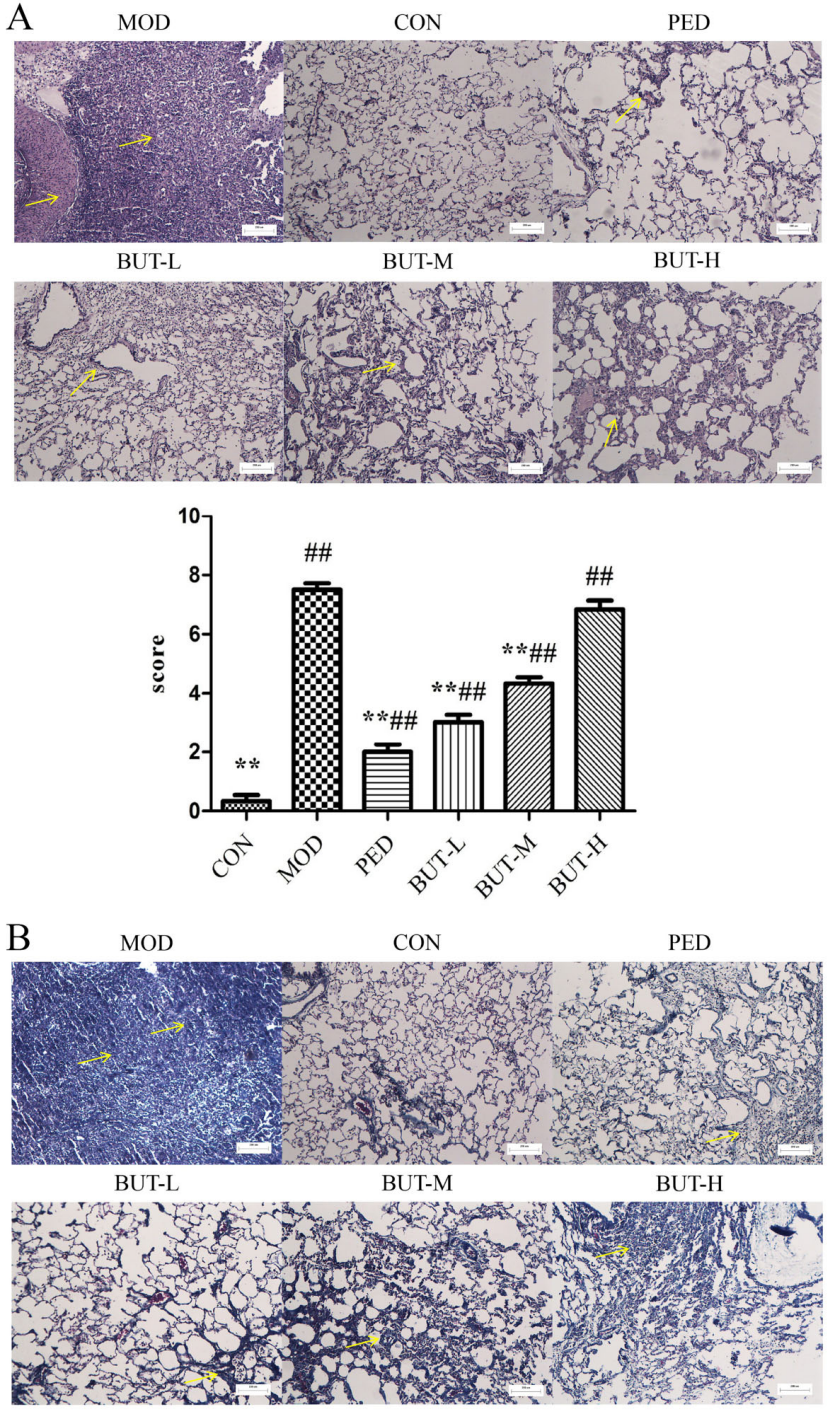
Pathological changes in the pulmonary tissue: **A**, Representative images of hematoxylin and eosin staining (×100, scale bar 200 μm) and score. **B**, Representative images of Masson staining (×100, scale bar 200 μm). Data are reported as means±SD. **P<0.01 compared to the model group; ^##^P<0.01 compared to the control group (ANOVA). CON: control; MOD: model; PED: prednisone; BUT-L: low-dose n-butanol extract of *Amygdalus mongolica*; BUT-M: medium-dose BUT; BUT-H: high-dose BUT.

The H&E-stained tissue images in Figure 1A show clear intrapulmonary structures with no thickening of the alveolar septa in the rat lung tissue of the CON group (Supplementary Table S1). In the MOD group, significant thickening of the alveolar septa, loss of alveolar structures, and formation of extensive foci of fibrosis were observed. Clear alveolar structure and significantly reduced lung fibrosis was observed in the PED and BUT groups compared to the MOD group. The histopathological scores indicated the degree of damage in each group, with the PED and BUT groups significantly alleviating PF in the rats, except in the BUT-H group.

The Masson-stained tissue images in Figure 1B show normal lung tissue structure with minor peri-alveolar or bronchial wall thickening. In the MOD group, fibrotic changes were visible in the lung tissue. A significant reduction in the alveolar or bronchial wall thickening, alveolar structural destruction, focal fibrosis formation, and pulmonary fibrosis degree was observed in the PED and BUT groups compared to the MOD group.

### Expression of factors related to the TGF-**β**1/Smads signaling pathway

**Figure 2:**
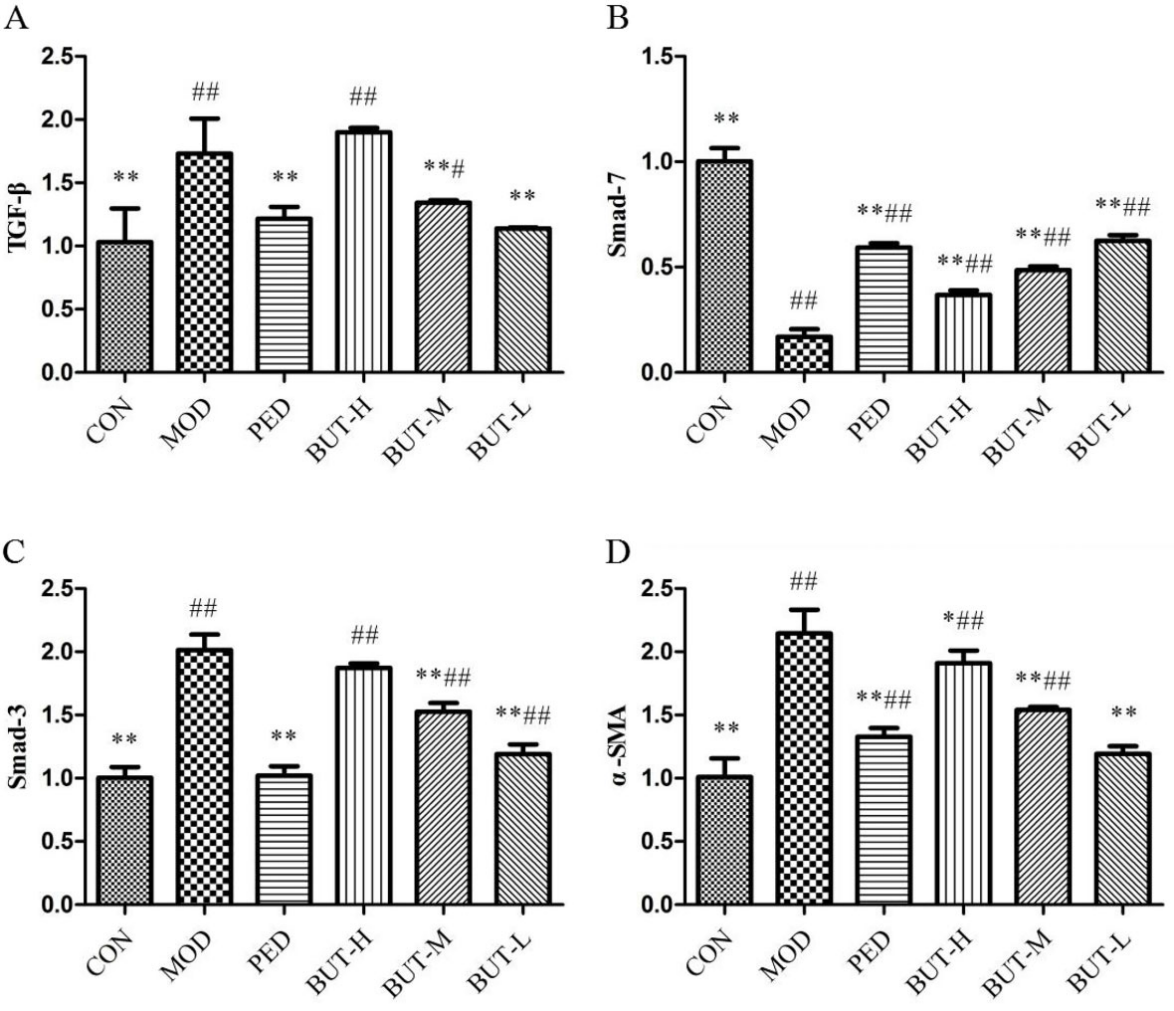
Effect of the n-butanol extract of *Amygdalus mongolica* (BUT) on the TGF-β1/Smad signal transduction pathway in rats with pulmonary fibrosis. Data are reported as means±SD. *P<0.05, **P<0.01 compared to the model group; ^#^P<0.05 and ^##^P<0.01 compared to the control group (ANOVA). CON: control; MOD: model; PED: prednisone; BUT-H: high-dose BUT; BUT-M: medium-dose BUT; BUT-L: low-dose BUT.

qRT-PCR revealed that the mRNA expression of TGF-β_1_, Smad-3, and α-SMA was significantly increased, whereas that of Smad-7 was significantly inhibited in the MOD group compared with the CON group. Furthermore, determination of the mRNA levels of TGF-β_1_, Smad-3, Smad-7, and α-SMA after PED and BUT treatments showed that the mRNA expression of Smad-7 was significantly increased, whereas that of TGF-β_1_, Smad-3, and α-SMA was significantly inhibited compared with the MOD group (P<0.05) (Figure 2 and Supplementary Table 2). Therefore, these results suggested that BUT inhibited oxidative stress and extracellular matrix deposition in the lung tissue, thereby affecting bleomycin-induced fibrotic production.

### Multivariate data analysis

**Figure 3:**
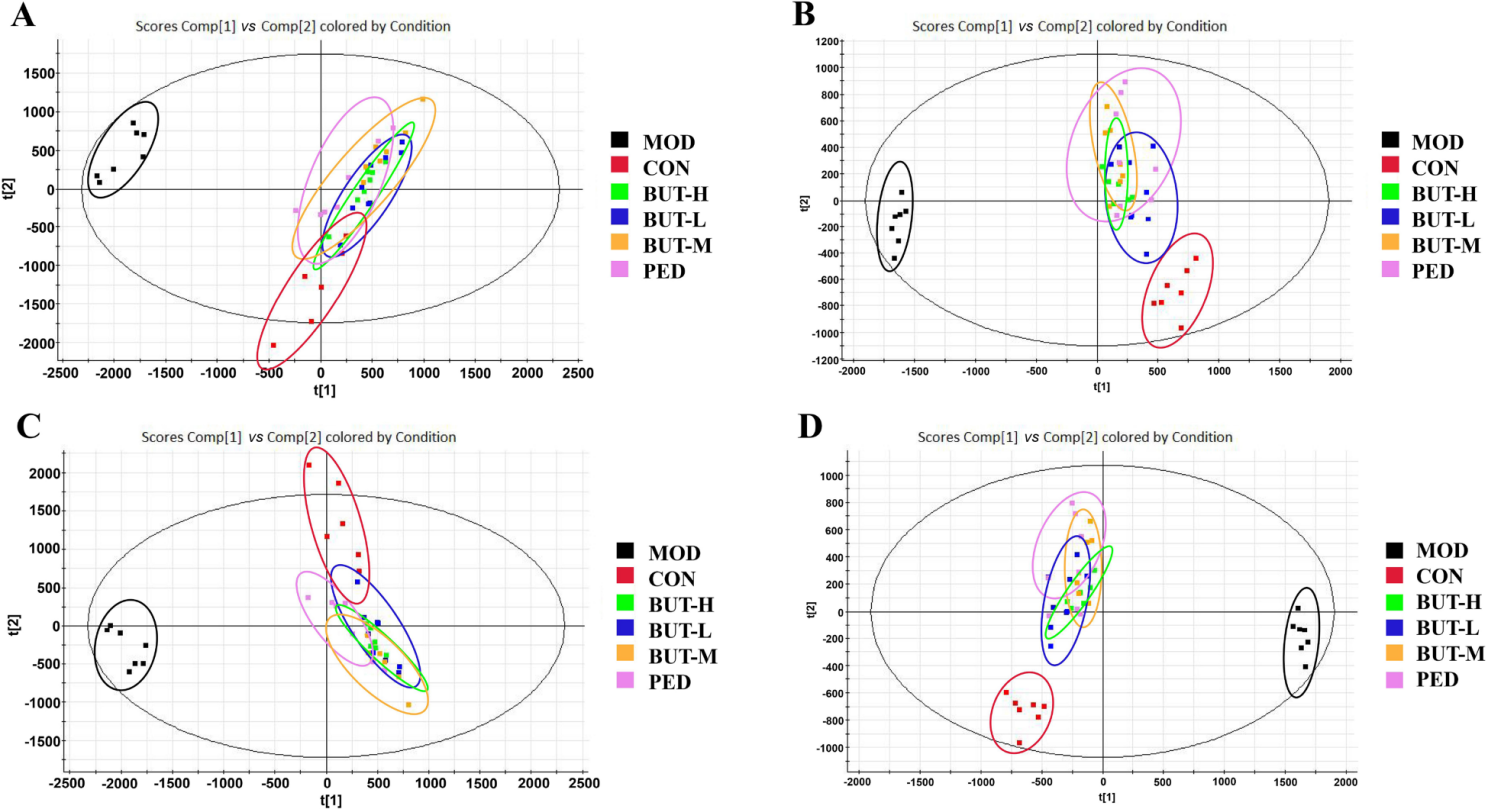
Principal component analysis (PCA) score plot and partial least squares discriminant analysis (PLS-DA) score of the control (CON), model (MOD), positive drug prednisone (PED) groups, high BUT dose (BUT-H), medium BUT dose (BUT-M), and low BUT dose (BUT-L) groups of the n-butanol extract of *Amygdalus mongolica* (BUT). **A**, PCA score plot in positive ion mode. **B**, PCA score plot in negative ion mode. **C**, PLS-DA score plot in positive ion mode. **D**, PLS-DA score plot in negative ion mode.

Unsupervised PCA of the original data was first performed (Figure 3A and B) showing a difference in clustering between the CON and MOD groups and similar clustering between the PED, BUT-H, BUT-M, BUT-L, and CON groups. Furthermore, PLS-DA was performed, and the data were again subjected to supervised metabolomic analyses, which showed a clear separation between the CON and MOD groups and a change in the metabolites of the MOD groups (Figure 3C and D), indicating that the PF model was successfully established. A further separation of the MOD group metabolites from the CON, PED, BUT-L, BUT-M, and BUT-H groups was observed. Moreover, the treatment groups of BUT-L and PED had similar results to the CON group, indicating the effect of BUT on rat metabolism.

### Screening and identification of potential biomarkers

**Figure 4:**
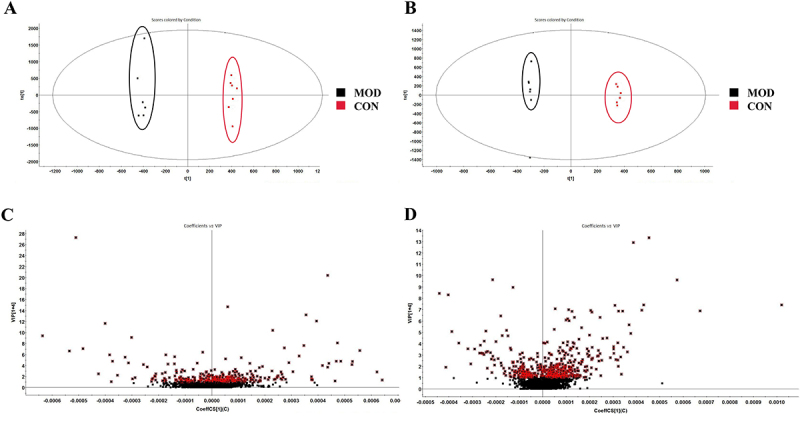
Orthogonal partial least squares discriminant analysis (OPLS-DA) score plot and Volcano plot map of the control (CON) and model (MOD) groups. **A**, OPLS-DA diagram in positive ion mode. **B**, OPLS-DA diagram in negative ion mode. **C**, Volcano plot map in positive ion mode. **D**, Volcano plot map in negative ion mode.

**Figure 5:**
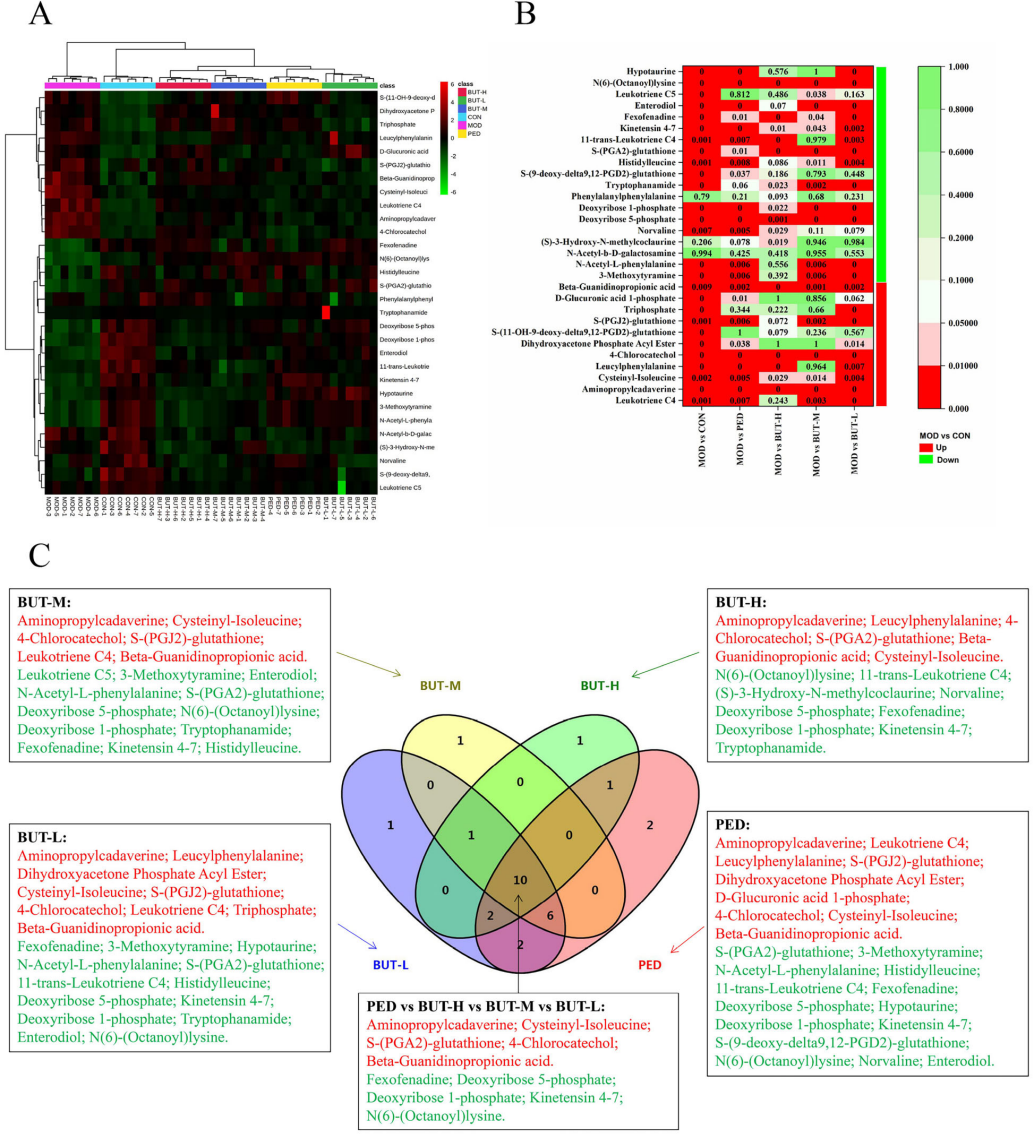
**A**, Heat map of the differentially abundant metabolites in all groups. The shades of red and green represent an increase and decrease in the expression of the corresponding genes, respectively. **B**, P-value heat map of the differentially abundant metabolites in all groups. The degree of color saturation indicates intergroup differences in metabolite expression values, with green and red indicating the non-significant and significant difference, respectively. **C**, Venn diagram of BUT-H, BUT-M, BUT-L, and positive drug prednisone (PED) biomarkers. Red and green colors indicate the up-regulated and down-regulated potential biomarkers, respectively, in the model group compared with the control group. CON: control; MOD: model; BUT-H: high-dose n-butanol extract of *Amygdalus mongolica*; BUT-M: medium-dose BUT; BUT-L: low-dose BUT.

To assess the difference between the CON and MOD groups, an orthogonal PLS-DA was performed based on minimal inter-class and intra-class differences (Figure 4A and B). The results showed no overlapping cases between the MOD and CON groups in the positive and negative ion modes, indicating the differences in metabolites between the two groups. A total of 30 differentially abundant metabolites (14 and 16 in positive and negative ion mode, respectively) were identified with adjusted VIP >1 and P<0.05 in the MOD group compared to that of the CON group (Figure 4C and D, Table 2). Furthermore, hierarchical clustering was performed for the sets of these differentially abundant metabolites, and the results are presented as a heat map (Figure 5A). Eleven metabolites were increased (red) and 19 were decreased (green) in the model group compared with the control group. Additionally, a heat map of the P-values was plotted to obtain concentration variations using ANOVA (Figure 5B). The Venn diagram (Figure 5C) shows potential biomarkers for significant callbacks in each group. PED, BUT-H, BUT-M, and BUT-L could respectively call back 23, 15, 18, and 22 potential biomarkers while simultaneously acting on 9 of the same potential biomarkers together. The BUT-M and BUT-L groups could jointly recall 10 potential biomarkers.

**Table 2:** Identification of potential biomarkers.

No.	m/z	RT	P value (*t*-test)	VIP	RankMS1hmdb	Common name	Trend (M *vs* C)
Positive ion mode							
1	142.16	12.99	3.57E-15	12.09	HMDB12189	Aminopropylcadaverine	↑
2	132.08	0.63	3.52E-04	2.24	HMDB13222	Beta-Guanidinopropionic acid	↑
3	190.09	5.41	4.77E-04	2.13	HMDB00022	3-Methoxytyramine	↓
4	190.09	5.41	4.77E-04	2.13	HMDB00512	N-Acetyl-L-phenylalanine	↓
5	244.08	0.63	1.83E-10	1.83	HMDB00853	N-Acetyl-b-D-galactosamine	↓
6	646.28	12.28	9.65E-11	1.78	HMDB12993	Leukotriene C5	↓
7	338.17	10.78	0.00E+00	1.77	HMDB06921	(S)-3-Hydroxy-N-methylcoclaurine	↓
8	118.09	0.65	1.90E-12	1.71	HMDB13716	Norvaline	↓
9	279.16	11.08	1.89E-12	1.56	HMDB13243	Leucylphenylalanine	↑
10	180.99	0.12	7.80E-11	1.34	HMDB11750	Dihydroxyacetone phosphate acyl ester	↑
11	237.02	0.05	3.69E-16	1.32	HMDB01031	Deoxyribose 5-phosphate	↓
12	237.02	0.05	3.69E-16	1.32	HMDB01351	Deoxyribose 1-phosphate	↓
13	625.28	3.69	3.00E-08	1.12	HMDB13302	Phenylalanylphenylalanine	↓
14	204.12	0.68	1.12E-04	1.07	HMDB13318	Tryptophanamide	↓
Negative ion mode							
1	640.29	12.23	2.03E-06	5.25	HMDB13058	S-(9-deoxy-delta9,12-PGD2)-glutathione	↓
2	581.32	10.23	5.19E-03	3.59	HMDB28889	Histidylleucine	↓
3	662.28	10.71	1.16E-03	3.33	HMDB13062	S-(PGA2)-glutathione	↓
4	624.29	10.77	1.16E-10	2.19	HMDB01198	Leukotriene C4	↑
5	624.29	10.77	1.16E-10	2.19	HMDB05095	11-trans-Leukotriene C4	↓
6	616.29	10.23	2.36E-03	2.14	HMDB12986	Kinetensin 4-7	↓
7	500.28	9.99	5.04E-10	2.08	HMDB05030	Fexofenadine	↓
8	649.3	10.24	3.95E-04	2.04	HMDB05056	Enterodiol	↓
9	293.18	8.69	2.80E-13	1.86	HMDB11684	N(6)-(Octanoyl)lysine	↓
10	217.03	0.66	1.74E-04	1.78	HMDB00965	Hypotaurine	↓
11	251.86	0.93	5.00E-07	1.46	HMDB03379	Triphosphate	↑
12	272.99	0.66	7.06E-07	1.35	HMDB03976	D-Glucuronic acid 1-phosphate	↑
13	286.97	3.22	2.15E-03	1.29	HMDB41810	4-Chlorocatechol	↑
14	640.29	11.93	5.84E-04	1.2	HMDB13063	S-(PGJ2)-glutathione	↑
15	255.08	4.46	0.00E+00	1.13	HMDB28778	Cysteinyl-Isoleucine	↑
16	664.29	11.46	1.83E-04	1.09	HMDB13056	S-(11-OH-9-deoxy-delta9,12-PGD2)-glutathione	↑

### Diagnostic potential of key metabolites

**Figure 6:**
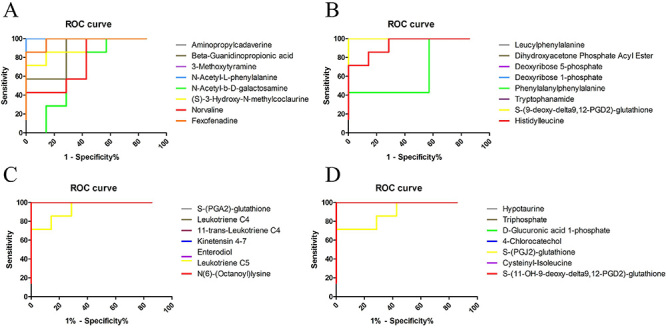
Receiver operating characteristic analysis of 30 potential biomarkers of pulmonary fibrosis.

ROC analyses (Figure 6 and Supplementary Table S3) were performed to further validate the diagnostic potential of the 30 biomarkers of PF. Area under the curve (AUC) reflects the quality of the ROC curve. The diagnostic accuracy of a biomarker was indicated by 0.7 < AUC <0.9, with AUC >0.9 indicating a high biomarker accuracy. The results revealed 28 potential biomarkers with AUC >0.7, except for N-acetyl-b-D-galactosamine and phenylalanylphenylalanine. The results showed that the 28 biomarkers had AUC values greater than 0.7 and were top-ranked biomarker candidates for the diagnosis of PF and objective indices for the evaluation of drug efficacy and prognosis.

### Analysis of related metabolic pathways and the screening of key biomarkers

**Figure 7:**
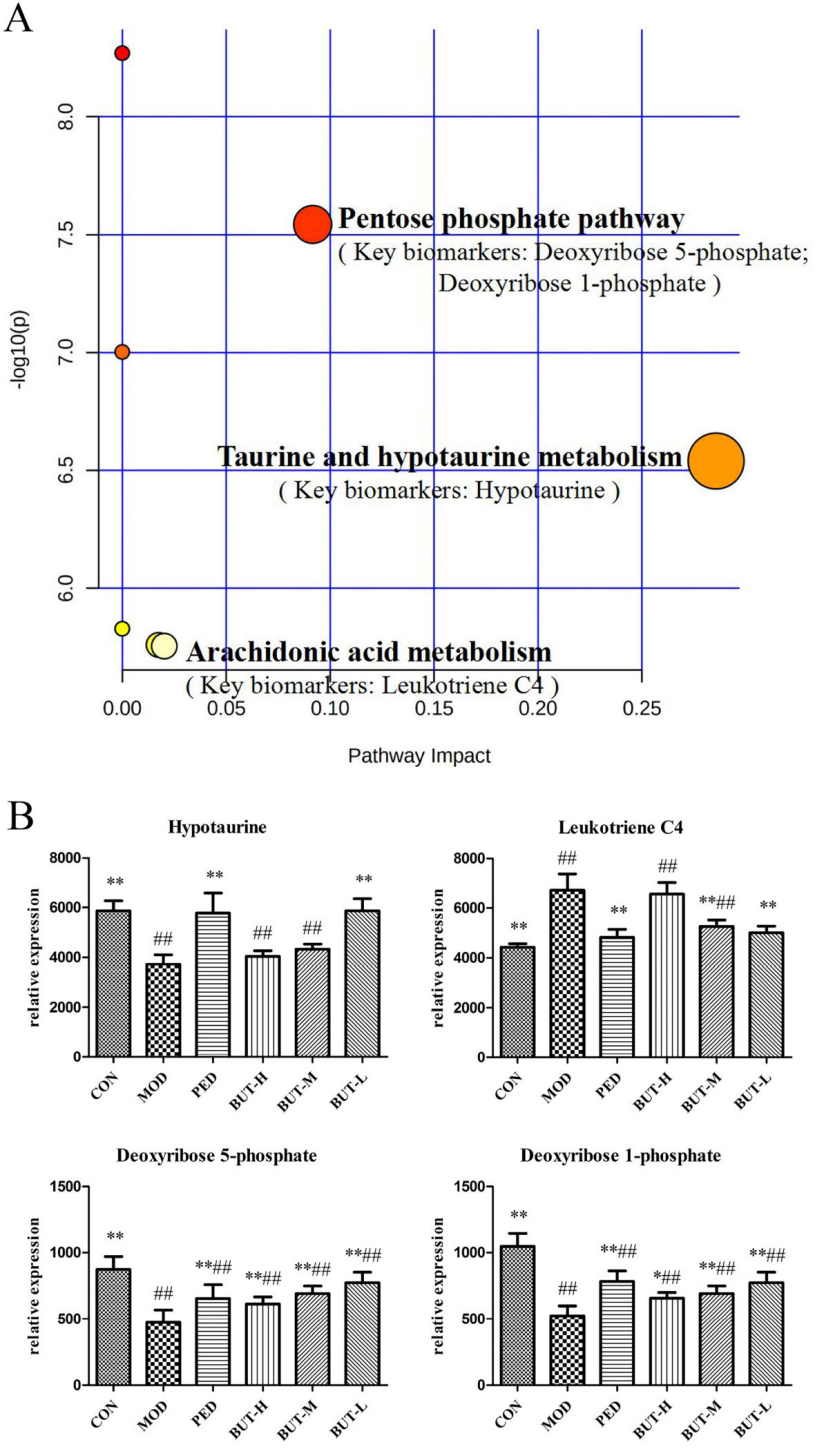
**A**, Metabolic pathway analyses. Summary of the altered metabolic pathways determined using MetPA and Metabo Analyst 4.0. The size and color of each circle indicate the pathway impact value and P-value, respectively. **B**, Effect of n-butanol extract on key biomarkers. Data are reported as means±SD. *P<0.05, **P<0.01 compared to the model group; ^##^P<0.01 compared to the control group (ANOVA). CON: control; MOD: model; PED: prednisone; BUT-H: high-dose n-butanol extract of *Amygdalus mongolica*; BUT-M: medium-dose BUT; BUT-L: low-dose BUT.

The 28 potential key biomarkers of PF were imported into the MetPA web-based tool to analyze the relevant pathways involved in the mechanism of BUT in PF, specifically its treatment. The most affected pathways were found to be the pentose phosphate pathway, taurine and hypotaurine metabolism, and arachidonic acid metabolism (impact value >0.02 and -log (*p*) >2) (Figure 7A). Finally, four crucial serum metabolites were identified from these pathways. To further explain the mechanism of BUT-mediated protection against PF, the relative concentrations of the four crucial biomarkers were calculated in all groups and are shown in Figure 7B. BUT-H, BUT-M, and BUT-L groups could significantly callback 2, 3, and 4 key biomarkers, respectively (Supplementary Table S4). The low BUT dose was effective on all three metabolic pathways and restored all four key biomarkers, indicating that it is the optimal dose for inducing antifibrotic effects.

## Discussion

The bleomycin-induced PF model is the most used and recognized animal model of PF (19,20). To probe the potential therapeutic value of BUT, different doses of BUT were assessed. A previous study reported that the effective dose range of *A. mongolica* BUT for its hypolipidemic and antioxidant action is 1.0-2.0 g/kg (8). Additionally, the content of amygdalin in BUT was reported to be 47.72% (8). Amygdalin has been used to treat different types of cancers and organ fibrosis (22). Therefore, based on the previously reported effective dose ranges, the doses used in this study were designed as follows: BUT-H, 1.75 g/kg; BUT-M, 1.5 g/kg; BUT-L, 1.25 g/kg.

**Figure 8:**
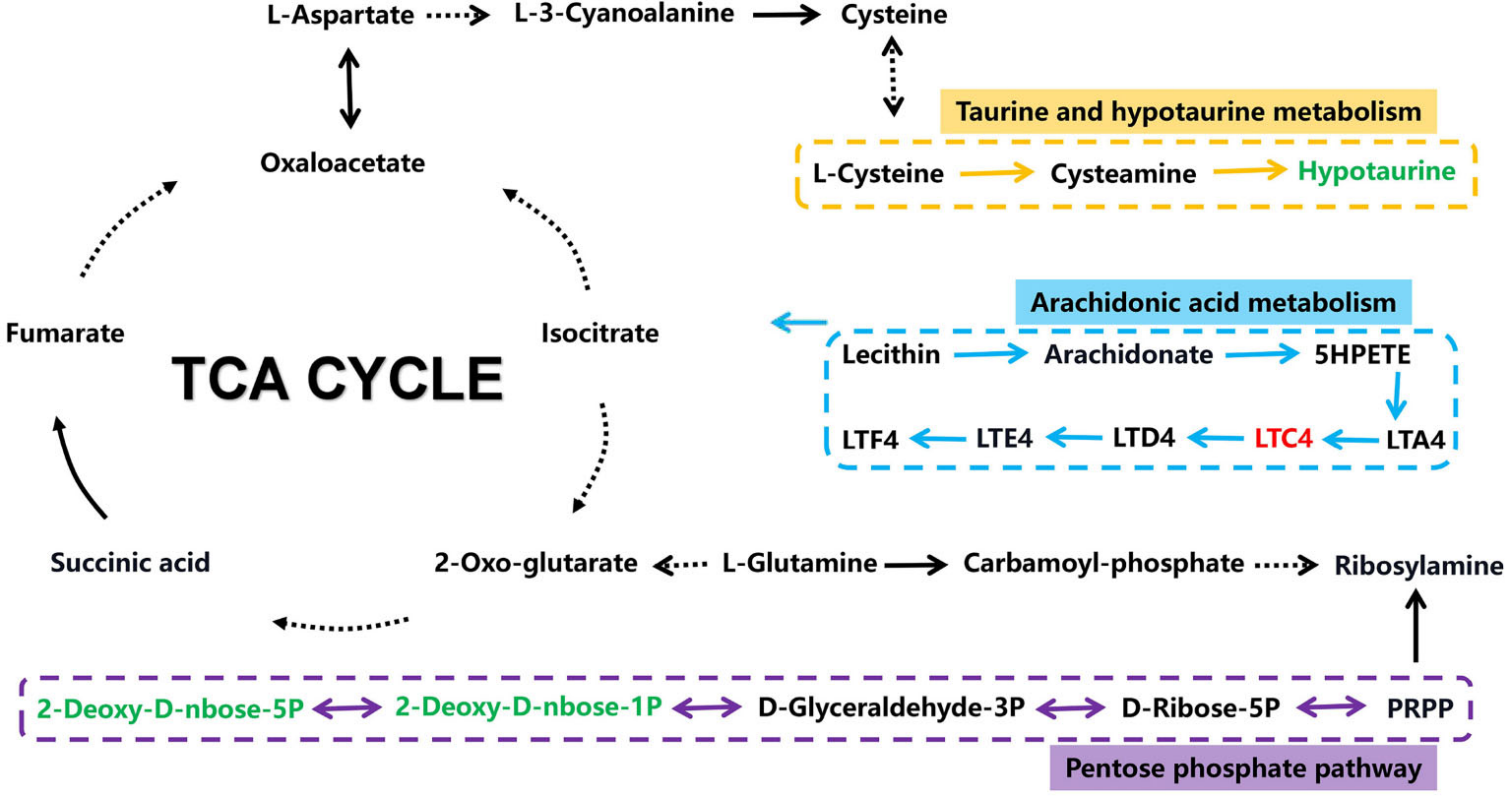
Potential targets and metabolic pathway networks of *Amygdalus mongolica* in anti-pulmonary fibrosis. Red and green colors represent the increased and decreased metabolite levels, respectively, in the model group compared with the control group.

In clinical settings, histopathological diagnosis is the gold standard for the diagnosis of PF, which directly reflects lung tissue injury and fibrosis degree (23). The histopathological findings showed that the alveolar structure was clear with a significant reduction in the alveolar or bronchial wall thickening, focal fibrosis formation, and PF degree in the PED group and BUT groups, compared to the MOD group, especially in the BUT-L and BUT-M groups.

Metabolomic analyses indicated that each dose of BUT, especially the low dose, could improve PF to varying degrees. BUT-L mainly targets taurine and hypotaurine metabolism, the citrate cycle, and the pentose phosphate pathway, which are affected during PF and lead to aberrant levels of hypotaurine, leukotriene C4, deoxyribose 5-phosphate, and deoxyribose1-phosphate dopamine (Figure 8).

The metabolism of cysteamine generates several intermediates, including hypotaurine and taurine (24). Fellman and Roth (25) reported that hypotaurine acts as a scavenger *in vivo*. Aruoma et al. (26) have also shown that hypotaurine has potent antioxidant effects *in vivo*. Another study demonstrated that taurine and nicotinic acid inhibit the activation of the nuclear factor-κ B pathway, thereby reducing the release of interleukin (IL)-1, IL-6, tumor necrosis factor-α, TGF-β inflammatory factor, and fibroblast cytokine (27). This study indicated that the low dose of BUT participated in taurine and hypotaurine metabolism by reducing the level of hypotaurine and inhibiting the release of TGF-β inflammatory factors and fibroblasts, which have anti-inflammatory and antioxidant functions, and thereby ameliorate the degree of PF.

Leukotriene C4 (LC4) is formed by the condensation of leukotriene A4 with a glutathione molecule via catalysis by glutathione S-transferase or leukotriene C4 synthase. It is an unsaturated fatty acid metabolite that plays a role in arachidonic acid metabolism (28). Beller et al. (29) found that cysteinyl leukotriene (Cys-LT) plays an important role in chronic pulmonary inflammation and fibrosis induced by bleomycin in mice. Moreover, LC4 synthase (the key enzyme of Cys-LT biosynthesis) was strongly associated with alveolar septum thickening and collagen deposition induced by macrophages and fibroblasts. Liu et al. (30) found that the recruitment of monocytes to the inflammatory sites by TGF-β enhanced the inflammatory response, wherein monocytes were induced to synthesize IL-1, IL-6, and LC4. Hirata et al. (31) reported that LC4 promotes TGF-β expression and fibrosis in bleomycin-induced PF in mice. Moreover, our study indicated that LC4 was a key biomarker of arachidonic acid metabolism and played an important metabolic role in rats with PF by reducing the level of LC4 and inhibiting the expression of TGF-β, thereby inducing anti-fibrosis, anti-inflammatory, and lung tissue-protective functions.

Deoxyribose (DR), a dephosphorylated product of 1-phosphate deoxyribose (DR-1-P), has angiogenic activity. Moreover, 5-phosphate deoxyribose (DR-5-P) and (DR-1-P) can convert into each other *in vivo*. At higher non-physiological concentrations, reducing sugar DR could induce the apoptosis of peripheral blood mononuclear cells (32) and fibroblasts (33). DR, DR-1-P, and DR-5-P are more likely to be formed via the action of phosphoribosyl (EC5.4.2.7) and are involved in the non-enzymatic glycosylation process (34). However, studies on the cellular metabolism of DR-1-P are scarce. DR-1-P has the potential to undergo dephosphorization and form DR to leave the cell. Conversely, it can be isomerized to DR-5-P by phosphate nuclear mutase and subsequently enter the glycolysis pathway (35). Previous studies have demonstrated that DR-1-P and DR-5-P up-regulate the protein expression of heme oxygenase-1 and integrin b3, which are two important markers of redox stress (36). Additionally, vascular endothelial growth factor, vascular endothelial growth factor receptor 2, IL-8, and stromal cell-derived factor 1a have similar effects. In this study, the low dose of BUT was shown to act on the pentose phosphate pathway by modulating the levels of DR-1-P and DR-5-P, thereby reducing oxidative stress and acting on fibroblasts and reducing the degree of PF.

TGF-β_1_ is a major fibrogenic factor that activates downstream fibrosis-related target genes to cause tissue fibrosis and tissue scarring (37). Smad proteins play a key role in the TGF-β_1_ signal transduction (38) by up-regulating the expression of α-SMA and collagen, which promotes fibroblast-myofibroblast transdifferentiation and extracellular matrix production that contribute to tissue fibrosis (39). α-SMA is an important contractile phenotype marker for myofibroblasts, which can contract, synthesize collagen, promote collagen I deposition, and subsequently induce fibrosis (15). Smad-3 protein directly binds to the DNA sequence of multiple profibrotic cytokines, whereas Smad-7 protein acts as an inhibitory protein. Overexpressed Smad-7 inhibits the phosphorylation of the TGF-β_1_ receptor, and the Smad-3 protein blocks the activation of the TGF-β_1_ signal (38). In this study, qRT-PCR was performed to validate the differential expression of factors related to the TGF-β_1_/Smads signaling pathway. Furthermore, the BUT-administered groups showed a reduction in the expression of TGF-β_1_, Smad-3, and α-SMA mRNA and an increase in the expression of Smad-7m RNA in the lung tissue compared to the MOD group, indicating that BUT could inhibit the production and deposition of the extracellular matrix via the TGF-β_1_/Smads signaling pathway. Therefore, the degree of PF could be ameliorated, which was further confirmed by the metabolomic analyses.

### Conclusions

BUT has been ascribed to have potent antifibrotic effects in PF. This study demonstrated that BUT inhibited PF and improved lung function by restoring four key biomarkers and three metabolic pathways, which could be attributed to the anti-inflammation and antioxidative effects and the inhibition of the fibrinogen release functions of these pathways. Two key biomarkers, hypotaurine and LC4, blocked the TGF-β/Smads signaling pathway. Furthermore, this study provided a foundation for the clinical application of *A. mongolica*.

## Supplementary Material

Click here to view [pdf].

## References

[1] Peng L, Wen L, Shi QF, Gao F, Huang B, Meng J (2020). Scutellarin ameliorates pulmonary fibrosis through inhibiting NF-κB/NLRP3-mediated epithelial-mesenchymal transition and inflammation. Cell Death Dis.

[2] Zhao X, Sun J, Chen Y, Su W, Shan H, Li Y (2018). lncRNA PFAR promotes lung fibroblast activation and fibrosis by targeting miR-138 to regulate the YAP1-twist axis. Mol Ther.

[3] Sun X, Yan H, Zhang Y, Wang X, Qin D, Yu J (2019). Preparative separation of diterpene lactones and flavones from andrographis paniculate using off-line two-dimensional high-speed counter-current chromatography. Molecules.

[4] Zhang Y, Lu P, Qin H, Zhang Y, Sun X, Song X (2021). Traditional Chinese medicine combined with pulmonary drug delivery system and idiopathic pulmonary fibrosis: Rationale and therapeutic potential. Biomed Pharmacother.

[5] Yuan YL (2007). Pharmacological action and clinical application of Rhizomaplumata (in Chinese). J Modern Med Health.

[6] Gao C, Bai WF, Zhou HB, Hao HM, Bai YC, Liu QL (2021). Metabolomic assessment of mechanisms underlying anti-renal fibrosis properties of petroleum ether extract from *Amygdalus mongolica*. Pharm Biol.

[7] Wang J, Zhou HB, Wu Tong, Wu PS, Liu QL, Shi SL (2021). Amygdalin isolated from *Amygdalus mongolica* protects against hepatic fibrosis in rats. Acta Pharm.

[8] Zhao YS, Wu PS, Zhou HW, Cheng XH, Shi SL, Li QL (2017). Studies on dose-effect relationship of n-butanol extracts *of Amygdalus mongolica* on reducing blood lipid and its chemical constituents (in Chinese). Sci Technol Food Ind.

[9] Du HK, Song FC, Zhou X, Li H, Zhang JP (2010). Effect of amygdalin on serum proteinic biomarker in pulmonary fibrosis of bleomycin-induced rat [in Chinese]. Zhonghua Lao Dong Wei Sheng Zhi Ye Bing Za Zhi.

[10] Guo J, Wu W, Sheng M, Yang S, Tan J (2013). Amygdalin inhibits renal fibrosis in chronic kidney disease. Mol Med Rep.

[11] Liu Q, Zhou HB, Shi SL, Liu QL, Gao C, Hao HM (2020). Protective effect of N-butanol extract of *Amygdalus mongolica* on renal fibrosis in rats (in Chinese). Chin Arch Trad Chin Med.

[B12] Wu T, Zhou HB, Wang J, Chang H, Bai WF, Quan BW (2021). Effect of different solvent extracts of *Amygdalus mongolica* on liver fibrosis rat models induced by carbon tetrachloride and its mechanisms (in Chinese). Sci Tech Food Indust.

[13] Quan BW, Wu T, Liu Q, Gao C, Zhou HB, Bai YC (2020). Protective effect of different polar parts of *Amygdalus mongolica* on pulmonary fibrosis rat models induced by bleomycin. Sci Tech Food Indust.

[14] Hasaneen NA, Cao J, Pulkoski-Gross A, Zucker S, Foda HD (2016). Extracellular Matrix Metalloproteinase Inducer (EMMPRIN) promotes lung fibroblast proliferation, survival and differentiation to myofibroblasts. Respir Res.

[15] Fan ML, Ying MF, Zhao R, Jing YC, Li MX (2020). Research progress on the role of TGF-β signaling pathway in fibrotic diseases. Med J Chin People's Liberation Army.

[16] Lu S, Yang SL, Rao Y, Feng YL (2018). Application of metabolomics and related technologies in research and development field of traditional Chinese medicine [in Chinese]. Zhongguo Zhong Yao Za Zhi.

[17] Gao C, Chang H, Zhou HB, Liu Q, Bai YC, Liu QL (2022). Metabolomics reveal the mechanism for anti-renal fibrosis effects of an n-butanol extract from *Amygdalus mongolica*. Acta Pharm.

[18] Chang H, Liu Q, Bai WF, Bai YC, Jia XY, Gao C (2020). Protective effects of *Amygdalus mongolica* on rats with renal fibrosis based on serum metabolomics. J Ethnopharmacol.

[19] Zhang DS, Ma CJ (2019). Methods and evaluation of animal models of induced pulmonary fibrosis (in Chinese). Acta Neuropharmacol.

[20] Wang P, Li TG (2020). Establishment of a mouse lung fibrosis model induced by paraquat poisoning (in Chinese). Pract Pharm and Clin Remed.

[21] Li XM, Peng JH, Sun ZL, Tian HJ, Duan XH, Liu L (2016). Chinese medicine CGA formula ameliorates DMN-induced liver fibrosis in rats via inhibiting MMP2/9, TIMP1/2 and the TGF-β/Smad signaling pathways. Acta Pharmacol Sin.

[22] Zhou C, Qian L, Ma H, Yu X, Zhang Y, Qu W (2012). Enhancement of amygdalin activated with β-D-glucosidase on HepG2 cells proliferation and apoptosis. Carbohydr Polym.

[23] Zhang W, Jiang LD, Zhang XM, Lu XF, Wu JJ, Deng J (2008). Influence of Feixian formula on lung quotiety and pulmonary pathological changes in rats with bleomycin induced pulmonary fibrosis (in Chinese). J Beijing Uni of Trad Chin Med.

[24] Barbieri D, Grassilli E, Monti D, Salvioli S, Franceschini MG, Franchini A (1994). D-ribose and deoxy-D-ribose induce apoptosis in human quiescent peripheral blood mononuclear cells. Biochem Biophys Res Commun.

[25] Fellman JH, Roth ES (1985). The biological oxidation of hypotaurine to taurine: hypotaurine as an antioxidant. Prog Clin Biol Res.

[26] Aruoma OI, Halliwell B, Hoey BM, Butler J (1988). The antioxidant action of taurine, hypotaurine and their metabolic precursors. Biochem J.

[27] Gurujeyalakshmi G, Wang Y, Giri SN (2000). Taurine and niacin block lung injury and fibrosis by down-regulating bleomycin-induced activation of transcription nuclear factor-kappaB in mice. J Pharmacol Exp Ther.

[28] Oga T, Handa T, Mishima M, Chin K, Narumiya SJI (2013). Roles of eicosanoids in pulmonary fibrosis. Inflamm Regen.

[29] Beller TC, Friend DS, Maekawa A, Lam BK, Austen KF, Kanaoka Y (2004). Cysteinyl leukotriene 1 receptor controls the severity of chronic pulmonary inflammation and fibrosis. Proc Natl Acad Sci USA.

[30] Liu R, Zhao QP, Dong HF, Jiang MS (2017). The TGF-β signaling pathways and their biological functions (in Chinese). J Pathogen Biol.

[31] Hirata H, Arima M, Fukushima Y, Sugiyama K, Tokuhisa T, Fukuda T (2013). Leukotriene C4 aggravates bleomycin-induced pulmonary fibrosis in mice. Respirology.

[32] Brunetti D, Dusi S, Giordano C, Lamperti C, Morbin M, Fugnanesi V (2013). Pantethine treatment is effective in recovering the disease phenotype induced by ketogenic diet in a pantothenate kinase-associated neurodegeneration mouse model. Brain.

[33] Kletsas D, Barbieri D, Stathakos D, Botti B, Bergamini S, Tomasi A (1998). The highly reducing sugar 2-deoxy-d-ribose induces apoptosis in human fibroblasts by reduced glutathione depletion and cytoskeletal disruption. Biochem Biophys Res Commun.

[34] Monnier VM (1990). Nonenzymatic glycosylation, the Maillard reaction and the aging process. J Gerontol.

[35] de Bruin M, Smid K, Laan AC, Noordhuis P, Fukushima M, Hoekman K (2003). Rapid disappearance of deoxyribose-1-phosphate in platelet derived endothelial cell growth factor/thymidine phosphorylase overexpressing cells. Biochem Biophys Res Commun.

[36] Vara D, Wheeler-Jones C, Mace K, Mellor H, Pula G (2014). 531 Platelet-derived deoxyribose-1-phosphate promotes endothelial cell motility and angiogenesis *in vivo* via redox stimulation. Cardiovasc Res.

[37] Wei Y, Kim TJ, Peng DH, Duan D, Gibbons DL, Yamauchi M (2017). Fibroblast-specific inhibition of TGF-β1 signaling attenuates lung and tumor fibrosis. J Clin Invest.

[38] Wang L, Ding T, Gong WL, Yang CH, Liu F (2019). Effective components of traditional Chinese medicine for regulating TGF/Beta1/Smads signaling pathway in hepatic fibrosis [in Chinese]. Zhongguo Zhong Yao Za Zhi.

[39] Sang XH, Wang H, You XF, Yang XY (2019). Research progresses of small molecule inhibitors targeting TGF-β and its receptors. Acta Pharm Sin.

